# BCAT1 promotes osteoclast maturation by regulating branched-chain amino acid metabolism

**DOI:** 10.1038/s12276-022-00775-3

**Published:** 2022-06-27

**Authors:** Miyeon Go, Eunji Shin, Seo Young Jang, Miso Nam, Geum-Sook Hwang, Soo Young Lee

**Affiliations:** 1 Department of Life Science, The Research Center for Cellular Homeostasis, Ewha Womans University, Seoul, 03760 Republic of Korea; 2 Integrated Metabolomics Research Group, Western Seoul Center, Korea Basic Science Institute, Seoul, 03759 Republic of Korea; 3 Department of Chemistry & Nanoscience, Ewha Womans University, Seoul, 03760 Republic of Korea

**Keywords:** Differentiation, Targeted bone remodelling

## Abstract

Branched-chain aminotransferase 1 (BCAT1) transfers the amine group on branched-chain amino acids (BCAAs) to alpha-ketoglutarate. This generates glutamate along with alpha-keto acids that are eventually oxidized to provide the cell with energy. BCAT1 thus plays a critical role in sustaining BCAA concentrations and availability as an energy source. Osteoclasts have high metabolic needs during differentiation. When we assessed the levels of amino acids in bone marrow macrophages (BMMs) that were undergoing receptor activator of nuclear factor κB ligand (RANKL)-induced osteoclast differentiation, we found that the BCAA levels steadily increase during this process. In vitro analyses then showed that all three BCAAs but especially valine were needed for osteoclast maturation. Moreover, selective inhibition of BCAT1 with gabapentin significantly reduced osteoclast maturation. Expression of enzymatically dead BCAT1 also abrogated osteoclast maturation. Importantly, gabapentin inhibited lipopolysaccharide (LPS)-induced bone loss of calvaria in mice. These findings suggest that BCAT1 could serve as a therapeutic target that dampens osteoclast formation.

## Osteoporosis: Targeting amino acid enzyme could strengthen bones

An examination of amino acid metabolism processes in bone yields a possible therapeutic target for osteoporosis and other bone diseases. The excessive generation of osteoclasts (bone-resorbing cells) is a key trigger for conditions such as osteoporosis, where bones become fragile and prone to fracturing. Branched-chain amino acids (BCAAs) are an essential energy source for cells, particularly during differentiation and maturation. South Korean researchers led by Soo Young Lee at Ewha Womans University and Geum-Sook Hwang at the Korea Basic Science Institute, both in Seoul, examined the role of BCAAs and the enzymes that process them in bone metabolism. They found that one such enzyme, BCAT1, directly regulates BCAA levels. Both BCAT1 and intracellular BCAA rose rapidly during osteoclast differentiation. Inhibition of BCAT1 activity in mice resulted in improved bone mass.

## Introduction

Bone-forming osteoblasts and bone-resorbing osteoclasts act together to maintain bone homeostasis. If the balance between these cells is broken, a variety of skeletal diseases can ensue^1,2^. One of these diseases is osteoporosis, which causes bone fragility and greatly elevates the risk of fracture. It is a major global cause of health problems. At present, most therapies for osteoporotic bone loss focus on reducing osteoclast differentiation or activity^3^.

Osteoclasts are giant multinucleated cells that require sufficient nutrients during differentiation to survive and mature^4–6^. Consequently, osteoclasts exhibit accelerated glucose metabolism at an early stage of their development^7^. However, it remains unclear whether other metabolites participate in osteoclast differentiation and function.

There are three branched-chain amino acids (BCAAs), namely, leucine, isoleucine, and valine. All are essential amino acids. Physiological and pathological models have shown that BCAAs can regulate muscle protein synthesis and the release of alanine and glutamine^8^. Moreover, by acting as nitrogen donors in the central nervous system, BCAAs help to synthesize neurotransmitter such as γ-aminobutyric acid^9^. In addition, recent studies have shown that BCAA catabolism is important for cancer proliferation in the lung, pancreas, and brain^10–13^. The first enzymes that process BCAAs are branched-chain amino acid aminotransferases (BCATs). BCATs regulate BCAA concentrations and catabolism. BCATs consist of two isoforms: BCAT1 is located in the cytosol while BCAT2 has a mitochondrial distribution. While BCAT2 is ubiquitously expressed, BCAT1 expression is limited to the brain, ovary, and placenta^14,15^. Both BCATs convert BCAAs into branched-chain α-keto acids (BCKAs) by transferring the amine group on BCAAs onto α-ketoglutarate; this generates glutamate. The BCKAs are then catabolized in the mitochondria into acetyl-CoA by several acyl-CoA dehydrogenases, after which they are oxidized in the tricarboxylic acid (TCA) cycle. Thus, BCAAs are also an important source of energy. Since BCAT transamination activity is reversible, the BCATs play a key role in regulating BCAA concentrations^16^.

The potential role of BCATs and BCAA in bone metabolism and bone disease has not been investigated previously. Here we report that our global metabolite profiling analysis showed that BCAA levels are increased during receptor activator of nuclear factor κB ligand (RANKL)-induced osteoclast differentiation. We then showed that by shaping BCAA metabolism, BCAT1 can regulate osteoclast differentiation and may be a therapeutic target.

## Materials and methods

### Mice and cells

All animal experiments were conducted with 6-week-old C57BL/6 male mice. All animal experiments were approved by the Institutional Animal Care and Use Committee of Ewha Laboratory Animal Genomics Center and were conducted in accordance with the appropriate guidelines. BMMs were prepared from the mice as previously described^17^. They were then cultured in alpha-minimum essential medium (alpha-MEM; Hyclone, South Logan UT, USA) supplemented with 10% fetal bovine serum (Hyclone) and 1% penicillin/streptomycin (Gibco, Grand Island, NY, USA).

### Reagents

Recombinant human macrophage-colony stimulating factor (M-CSF) and RANKL were purchased from R&D systems (Minneapolis, MN, USA) and PeproTech EC Ltd. (London, UK) respectively. The TRAP staining kit was purchased from FUJIFILM Wako Pure Chemical Corporation (Tokyo, Japan). Lipopolysaccharide (LPS), BCAAs (L-leucine, L-isoleucine, L-valine), and BCKAs [KIC (4-Methyl-2-oxovaleric acid), KMV ((±)-3-Methyl-2-oxovaleric acid sodium salt) and KIV (Sodium 3-methyl-2-oxobutyrate)] were purchased from Sigma-Aldrich (St. Louis, Missouri, USA). Gabapentin was purchased from Santa Cruz Biotechnology (Dallas, TX, USA). The following antibodies were used in western blotting: anti-ERK, anti-phospho-ERK (T202/Y204), anti-p38, anti-phospho-p38 (T180/Y182), anti-JNK, anti-phospho-JNK (T183/Y185), anti-AKT, anti-phospho-AKT (S473), anti-p65, anti-phospho-p65 (Y536), anti-IκBα (Cell Signaling Technology, Danvers, MA, USA), anti-β-Actin, anti-NFATc1 (Santa Cruz Biotechnology), anti-BCAT1 (Novus Biologicals, Centennial, CO, USA), anti-BCAT2 (Abcam, Cambridge, UK) and anti-FLAG (Sigma-Aldrich). The anti-Atp6v0d2 antibody was provided by Y. Choi (University of Pennsylvania, Philadelphia PA, USA).

### In vitro osteoclast differentiation, culture with BCAAs or BCKAs and TRAP staining

BMMs were cultured in custom alpha-MEM that lacked leucine, isoleucine and valine (WELGENE, Gyeongsan, Korea) and contain 10% dialyzed fetal bovine serum (Gibco) and 1% penicillin/streptomycin (Gibco). BCAAs or BCKAs were added at various concentrations. To induce osteoclast differentiation, BMMs were treated with 30 ng/ml M-CSF and 100 ng/ml RANKL. In all experiments, the medium was removed and replaced with fresh medium every day. To determine the stage of osteoclast differentiation, the cells were fixed with 4% formaldehyde for 10 min and then stained for tartrate-resistant acid phosphatase (TRAP) at 37°C for 40 min by using the TRAP staining kit (FUJIFILM) according to manufacturer instructions. The TRAP^+^ multinucleated cells (defined as osteoclasts that were ≥200 μm in diameter) in nine microscopic fields (10× objective; magnification: ×100) in each of three wells of a 48-well plate were counted. The experiment was repeated three times. All images were obtained with an Olympus CKX53 inverted microscope (Olympus Corporation, Tokyo, Japan) with ToupTek Cam (ToupTek Photonics, Hangzhou, Zhejiang, P.R. China).

### Measurement of metabolites

BMMs were induced to undergo osteoclast differentiation as described above for the indicated times (0, 8, 24, 48 h) and intracellular metabolites were extracted by using a methanol/water/CHCl_3_ mixture (1/1.6/1, v/v/v). The aqueous supernatant containing the water-soluble metabolites was vacuum dried and resuspended in 600 μl of 0.1 M sodium phosphate-buffered deuterium oxide (pH 7.0) containing 0.1 mM 3-(trimethylsilyl) propionic-2,2,3,3-d_4_ acid (TSP-d_4_) (Sigma Aldrich). To measure BCAA uptake, osteoclasts were treated with M-CSF alone or RANKL alone for 7 days, after which the supernatant was harvested into a EP tube. The extracellular metabolites were extracted by adding 2 ml of methanol to 500 ml of culture media, followed by drying under nitrogen gas. The medium extract was reconstituted into 600 μl of 0.1 M sodium phosphate-buffered deuterium oxide (pH 7.0) containing 0.3 mM TSP-d_4_. Nuclear magnetic resonance (NMR) spectroscopy was conducted with an Ascend 800-MHz NMR with a triple-resonance 5 mm cryogenic probe (Bruker BioSpin AG) to obtain ^1^H-NMR spectra. The NMR data were processed with TopSpin Version 3.1 (Bruker Biospin, Rheinstetten, Germany). All spectra were phase and baseline-corrected. The metabolites were identified by using Chenomx NMR suite version 7.1 (Chenomx Inc., Edmonton, AB, Canada) and with spiking experiments to confirm the overlapping or slightly shifted peaks. The metabolites were quantified by using the 800 MHz library from Chenomx NMR Suite version 7.1, which employs the concentration of the TSP-d_4_ reference signal to determine the concentrations of the individual metabolites.

### Western blot analysis

Western blotting was conducted as described previously^18^. Briefly, cells were lysed using cell lysis buffer containing protease and phosphatase inhibitors, after which the proteins were boiled with SDS loading buffer, separated on SDS-polyacrylamide gels, electrophoretically transferred onto PVDF membranes (Millipore, Burlington, MA, USA), and probed with the indicated antibodies at a final dilution of 1:1000. Proteins were detected by using an enhanced chemiluminescence (ECL) detection kit (Bio-Rad Laboratories, Hercules, CA, USA).

### Real-time quantitative polymerase chain reaction

Total RNA was extracted using TRIzol (iNtRON Biotechnology, Seongnam, Korea) and then reverse-transcribed into cDNA by using a Diastar Reverse Transcription kit (BioFACT, Daejeon, Korea) according to manufacturer instructions. Real-time quantitative polymerase chain reactions (RT-qPCR) were conducted by using a SensiFAST™ SYBR® Hi-ROX Kit *(*Bioline Ltd., London, UK). The Applied Biosystems® StepOne™ Real-Time PCR Systems (Applied Biosystems, Foster City, CA, USA) was used for detection. The following gene-specific primers for PCR were used: Bcat1 sense, 5ʹ-GAAGTGGCGGAGACTTTTAGG-3ʹ, antisense, 5ʹ-TGGTCAGTAAACGTAGCTCCA-3ʹ; Bcat2 sense, 5ʹ-CAAAGGTGGAGACCAGCAGGTA-3ʹ, antisense, 5ʹ-TGGCGGATACACTCCAACAGCT-3ʹ; Nfatc1 sense, 5ʹ-CCAGAAAATAACATGCGAGCC-3ʹ, antisense, 5ʹ-GTGGGATGTGAACTCGGAAG-3ʹ; Atp6v0d2 sense, 5ʹ-CAGAGATGGAAGCTGTCAACATTG-3ʹ, antisense, 5ʹ-TGCCAAATGAGTTCAGAGTG-3ʹ; Dcstamp sense, 5ʹ-TGGAAGTTCACTTGAAACTACGTG-3ʹ, antisense, 5ʹ-CTCGGTTTCCCGTCAGCCTCTCTC-3ʹ; Itgav sense, 5ʹ-AACATCACCTGGGGCATTCA-3ʹ, antisense, 5ʹ-CGTCAGTGTGGGCGAAGTAA-3ʹ; Cd9 sense, 5ʹ-CTTGCTATTGGACTATGGCT-3ʹ, antisense, 5ʹ-GTCCGAGATAAACTGCTCCA-3ʹ; Mmp9 sense, 5′-GCCCTGGAACTCACACGACA-3′, antisense, 5′-TTGGAAACTCACACGCCAGAAG-3′; Ctsk sense, 5′-GGAAGAAGACTCACCAGAAGC-3′, antisense, 5′-GCTATATAGCCGCCTCCACAG-3′; Actin sense, 5ʹ-AGATGTGGATCAGCAAGCAG-3ʹ, antisense, 5ʹ-GCGCAAGTTAGGTTTTGTCA-3ʹ. The data were normalized to β-actin mRNA expression.

### Gene knockdown assay using siRNA

BMMs were transfected with 10 nM of small interfering RNAs (siRNA) for 5 h by using Lipofectamine™ RNAiMAX Transfection Reagent (Invitrogen, Waltham, MA, United States) according to manufacturers instructions. Serum-free opti-MEM (Gibco) was used to dilute the siRNA reagents. Si-Bcat2 and si-Control oligo were purchased from OriGene (Rockville, MD, USA). The mouse Bcat2 siRNA sequence was 5ʹ-GGAGUAGUUCGACAAAGUCUGCUGG-3ʹ. After 24 h, the cells were cultured with M-CSF (30 ng/ml) and RANKL (100 ng/ml) to induce their differentiation into osteoclasts.

### MTT assay

The MTT cell viability assay was used to identify the non-cytotoxic concentrations of gabapentin. BMMs were seeded in 96-well plates at a density of 1 × 10^6^ cells/well and incubated for 2 days with 2–20 mM gabapentin in the presence of 30 ng/ml M-CSF. Subsequently, 20 μl MTT [3-(4,5-dimethylthiazol-2-yl)2,5-diphenyltetrazolium bromide] (Sigma Aldrich) was added to each well. After incubation for 3.5 h at 37 °C, 150 μl MTT solvent was added to end the reaction and extract the formazan crystals. Absorbance at 570 nm was measured. Cell viability was calculated relative to control values.

### Gabapentin inhibition assay

BMMs were cultured with M-CSF (30 ng/ml) and RANKL (100 ng/ml) for 3 days with 2–10 mM gabapentin. The cells were stained with TRAP or subjected to the bone resorption assay described below. Alternatively, serum-starved BMMs were preincubated with 5 mM gabapentin before being stimulated with M-CSF (30 ng/ml) or RANKL (100 ng/ml) for 0–30 min. Western blot was then conducted.

### Bone resorption assay

Pre-osteoclasts were obtained by M-CSF (30 ng/ml) and RANKL (100 ng/ml) treatment for 2 days, and then transferred onto dentin slices (Immunodiagnostic Systems Ltd., Boldon Colliery, UK) in 96-well plates at a density of 2 × 10^4^ cells/well. Three days after the cells achieved a fully differentiated state, they were removed from the dentin slices. The dentin slices were stained with hematoxylin solution and the pits caused by osteoclast resorption were visualized by using an Olympus CKX53 inverted microscope with ToupTek Cam. Their area was quantified with Image-Pro Plus software (Media Cybernetic, Rockville, MD, USA).

### Plasmids and preparation of retrovirus

pMX-puro vector and Plat-E cells were kindly provided by T. Kitamura (University of Tokyo, Japan). The pCMV6-entry-mouse Bcat1 (Myc-DDK-tagged) cDNA clone (NM_007532) was purchased from OriGene Technologies.

The coding region of Bcat1 was introduced into the pMX-puro vector and a 3x-Flag epitope was placed at the end of the Bcat1 gene (pMX-puro-3x-flag-mBCAT1). A C338A mutant construct was generated by using site-directed mutagenesis. The following primers were used for mutagenesis: sense, 5ʹ-ACAGCCTGCGTTGTCGCCCCAGTCTCTGATATT-3ʹ, antisense, 5ʹ-AATATCAGAGACTG GGGCGACAACGCAGGCTGT-3ʹ.

Plat-E cells were transfected with empty pMX-puro vector, pMX-puro-Flag-BCAT1 or pMX-puro-Flag-BCAT1-C338A by using polyethylenimine (Sigma-Aldrich). BMMs were infected for 5–6 h with the viral supernatant collected from Plat-E cells. After 24 h, the infected BMMs underwent puromycin selection (2 μg/ml) for 2 days. The selected cells then further cultured with M-CSF (30 ng/ml) and RANKL (100 ng/ml) to induce their differentiation into osteoclasts.

### LPS-induced bone destruction model

The calvaria of 5-week-old mice were injected twice with a 48 h interval with the following solutions: phosphate-buffered saline (PBS group, *n* = 3), 12.5 mg/kg body weight LPS (LPS-alone group, *n* = 5), LPS plus 30 mg/kg body weight gabapentin (LPS + 30-gaba group, *n* = 5), LPS plus 100 mg/kg body weight gabapentin (LPS + 100-gaba group, *n* = 4). The mice were sacrificed 3 days after the last injection and the calvarial bones were harvested, fixed with 10% formalin for 16 h and then decalcified for 7 days in 0.5 M ethylenediaminetetraacetic acid. The decalcified bones were embedded in paraffin and cut into 5μm-thick sections that were stained for TRAP. Images were obtained by using an Olympus BX51 microscope (Olympus Corporation) with Olympus DP72 camera (Olympus Corporation) The images were analyzed by using OsteoMeasureXP (OsteoMetrics, Inc., Decatur, GA, USA).

### Statistical analysis

The data of one of ≥3 independent representative experiments are presented as mean ± SD. Groups were compared by a Student’s two-tailed *t*-test or two-way ANOVA. GraphPad Prism software (San Diego, CA, USA) was used for all analyses. *P* values of <0.05 were considered to indicate statistical significance. The *p* values were designated as *p ≤ 0.05; **p ≤ 0.01; ***p ≤ 0.001; ****p ≤ 0.0001; and N.S., not significant (*p* > 0.05).

## Results

### Intracellular BCAA levels and BCAA uptake rise during osteoclast differentiation

**Fig. 1:**
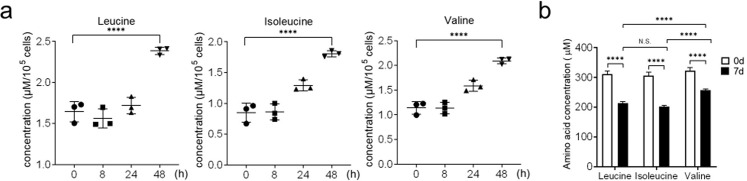
Intracellular BCAA levels and BCAA uptake from the culture medium both rise during osteoclast differentiation. **a**, **b** Changes in intracellular BCAAs (L-Leucine, L-Isoleucine, and L-Valine) levels (**a**) and BCAA uptake (**b**) during RANKL-induced osteoclast differentiation. BMMs were stimulated with M-CSF (30 ng/ml) and RANKL (100 ng/ml) for the indicated times. The cells (**a**) or their supernatants (**b**) were subjected to NMR spectroscopy to quantify the BCAAs. For **b**, net uptake was the difference between the BCAA control and test wells. ****p ≤ 0.0001 (*n* = 8).

To determine which metabolites are utilized during osteoclast differentiation, we subjected BMM to RANKL-induced osteoclast differentiation for the indicated times. The intracellular contents of the resulting pre-osteoclasts were then subjected to NMR spectroscopic analysis to quantify the metabolites that relate to energy production. These metabolites included amino acids, nucleotides and their derivatives, organic acids and amines, ketones, aldehydes and alcohols (Supplementary Fig. 1). During RANKL-induced differentiation, the intracellular levels of the three BCAAs rose in a time-dependent manner (Fig. 1a). The uptake of the BCAAs in the culture medium by the cells also increased over time. In three BCAAs, valine displayed the lowest uptake (Fig. 1b).

### BCAAs are needed for later stages of osteoclast differentiation

**Fig. 2:**
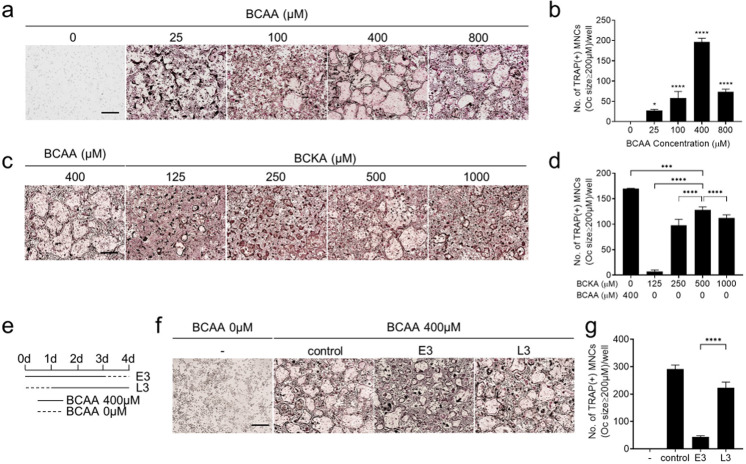
BCAAs are needed for the mid-late phase of osteoclast differentiation and can be replaced with BCKAs. BMMs were differentiated into osteoclasts with M-CSF (30 ng/ml) and RANKL (100 ng/ml) in custom medium at the indicated concentration of BCAAs (**a**, **b**) or BCKAs (**c**, **d**). **e**–**g** As depicted schematically in **e**, 400 μM BCAAs were added at the start of RANKL treatment on day 0 and then withdrawn on day 4 (E3) or added at day 1 and maintained until the end of the culture (L3) (**f**, **g**). At the end of the culture period, the cells were stained with TRAP, and the TRAP^+^ cells with diameters of ≥200 μm were counted. *p ≤ 0.05, ***p ≤ 0.001, ****p ≤ 0.0001 (*n* = 6). Scale bar, 500 μm.

To determine whether the BCAAs can affect osteoclast differentiation, BMMs were subjected to osteoclast differentiation with RANKL in a custom medium with different doses of BCAAs (0–800 μM). 400 μM BCAA induced the greatest number of giant multinucleated osteoclasts. Fewer such osteoclasts were observed with 25, 100, and 800 μM BCAAs. (Fig. 2a–b).

Given that BCAAs are catabolized into BCKAs in vivo^16^, we speculated that replacing leucine, isoleucine, and valine with the corresponding BCKAs in the osteoclast cultures described above could compensate for the lack of BCAAs. Thus, three BCKAs were added instead of BCAAs at concentrations ranging from 0 to 1000 μM. 500 μM BCKAs induced similar numbers of TRAP+ osteoclasts as 400 μM BCAAs (Fig. 2c, d).

To identify the osteoclast stages that are affected by BCAA levels during differentiation, BMMs were cultured in a medium with 400 μM BCAAs only during the early (E3) or the late (L3) three days (Fig. 2e). The absence of BCAAs on day 4 (E3) significantly decreased osteoclast maturation whereas the absence of BCAAs on day 1 (L3) did not markedly affect TRAP^+^ multinucleated osteoclast cell numbers (Fig. 2f, g). Thus, while BCAAs are not required initially for the formation of mature osteoclasts, they must be present during the late stages of osteoclast differentiation.

### Valine is more important for osteoclast maturation than the other BCAAs

**Fig. 3:**
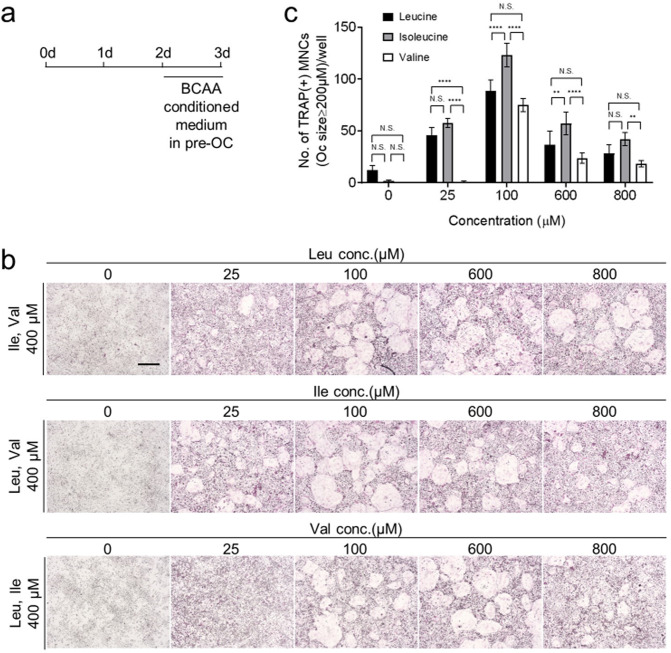
Valine is the most important BCAA for mid-late osteoclast differentiation. **a**–**c** As depicted schematically in **a**, pre-osteoclasts were generated in a medium containing 400 μM BCAAs by M-CSF (30 ng/ml) and RANKL (100 ng/ml), after which the medium was replaced for 1 day with custom medium containing 0, 25, 100, 600 or 800 μM of one of the three BCAAs and 400 μM of the remaining two BCAAs. The cells were fixed and stained for TRAP. TRAP^+^ cells with a diameter of ≥200 μm were counted (**c**). **p ≤ 0.01, ****p ≤ 0.0001 (*n* = 6). Scale bar, 500 μm.

To determine which of the three BCAAs is most important for osteoclast differentiation, BMMs were incubated with a medium containing 400 μM BCAAs for the first 2 of 3 days. On the third day, the medium was replaced with custom medium containing 0–800 μM of one of the three BCAAs; the remaining two BCAAs were also present at 400 μM concentrations (Fig. 3a). The absence of any one of the three BCAAs on day 3 severely hampered the differentiation of the pre-osteoclasts into multinucleate osteoclasts. Similarly, 25 μM of valine was associated with impaired differentiation; by contrast, 25 μM of leucine or isoleucine yielded considerable differentiation (Fig. 3b, c). Thus, while all three BCAAs must be present to induce osteoclast maturation, this process is more dependent on valine than leucine or isoleucine.

### The BCAT1 inhibitor gabapentin suppresses osteoclast differentiation

**Fig. 4:**
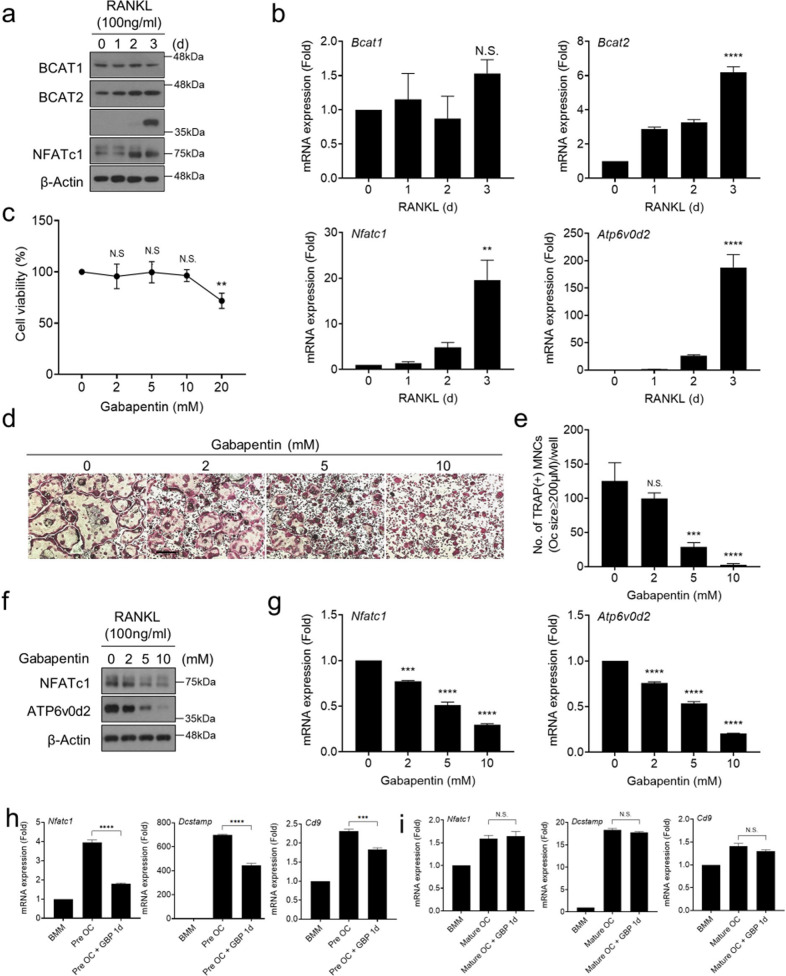
The BCAT1 inhibitor gabapentin inhibits osteoclast differentiation. **a**, **b** Effect of osteoclast differentiation on BCAT expression. BMMs were stimulated with M-CSF (30 ng/ml) and RANKL (100 ng/ml) for the indicated times. The cells were harvested and their lysates were subjected to (**a**) western blot or (**b**) RT-qPCR analysis of the indicated molecules. **c**–**g** Effect of gabapentin on osteoclast differentiation (**d**, **e**) and associated NFATc1 and ATP6v0d2 expression (**f**, **g**). BMMs were stimulated with M-CSF (30 ng/ml) and gabapentin at doses ranging from 0 to 20 mM for 3 days, after which the MTT cell viability assay was conducted (**c**). BMMs were incubated with M-CSF (30 ng/ml) and RANKL (100 ng/ml) with or without gabapentin at the indicated doses for 3 days. The cells were fixed, stained for TRAP (**d**) and the TRAP^+^ cells with a diameter of ≥200 μm were counted (**e**). Scale bar, 500 μm. Alternatively, the cells were subjected to western blot (**f**) or RT-qPCR (**g**) analysis of NFATc1 and ATP6v0d2 expression. β-actin antibodies served as the control in (**f**). **h**, **i** BMMs were incubated with M-CSF (30 ng/ml) and RANKL (100 ng/ml) for 2 days (**h**) and 4 days (**i**) to respectively generate pre-osteoclasts and mature osteoclasts. The cells were then treated with gabapentin (5 mM) for 1 day, harvested, lysed and were subjected to RT-qPCR analysis of the indicated molecules. *p ≤ 0.05, **p ≤ 0.01, ***p ≤ 0.001, ****p ≤ 0.0001 versus control (*n* = 4).

Since BCAT1 and BCAT2 are the first enzymes to metabolize BCAAs, we asked whether they played an important role in osteoclast differentiation. First, we examined their expression over time in BMMs that were stimulated with RANKL. As shown by western blot and RT-qPCR analysis, both BCATs were expressed throughout the differentiation process. The BCAT1 levels did not change after RANKL was introduced. By contrast, like the osteoclast markers ATP6v0d2 and NFATc1^19,20^, BCAT2 expression was steadily upregulated (Fig. 4a, b).

We then asked whether BCAT2 or BCAT1 affected osteoclast differentiation by siRNA knockdown and gabapentin mediated inhibition, respectively. Gabapentin is an anticonvulsant drug that is known to inhibit BCAT1^21^. First, siRNA-transfected BMMs were cultured to assess differentiation and resorption ability (Supplementary Fig. 2a). We observed that BCAT2 knockdown had no effect on either osteoclast differentiation or bone resorption activity (Supplementary Fig. 2b–e). We then conducted the gabapentin assay. To do so, we first identified the non-cytotoxic gabapentin dose range (2–10 mM) with the MTT cell viability assay (Fig. 4c). BMMs were then treated with gabapentin in the above dose range together with RANKL. Gabapentin prevented osteoclast maturation in a dose-dependent manner (Fig. 4d–e) but did not affect the bone resorption activity of the osteoclasts (Supplementary Fig. 3a–b). Gabapentin also did not affect the expression of the bone resorption markers *Ctsk* and *Mmp9* (Supplementary Fig. 3c).

To determine whether gabapentin blocked osteoclast differentiation in early signaling, the M-CSF and RANKL-induced signaling pathways (i.e., NF-κB and MAP kinases) were examined. Gabapentin did not affect both signaling pathways in the early stages (Supplementary Fig. 4) but did decrease the RANKL-stimulated NFATc1 and ATP6v0d2 mRNA and protein levels in a dose-dependent manner (Fig. 4f–g). Thus, BCAT1, but not BCAT2, is involved in osteoclast differentiation in mid-late sgnaling stages.

To further investigate the effect of gabapentin on specific processes that take place in the late stage of osteoclast differentiation, pre-osteoclasts (Fig. 4h) or mature osteoclasts (Fig. 4i) were treated gabapentin for 1 day and their mRNA expression of *Dcstamp*^22^ and *Cd9*^23^, two well-known osteoclast fusion markers, were measured. Gabapentin decreased both fusion markers in pre-osteoclasts but not mature osteoclasts. Since gabapentin treatment also suppressed the expression of *Nfatc1* in pre-osteoclasts (Fig. 4f–g), it appears that gabapentin may control osteoclast fusion by regulating *Nfatc1* expression.

### Expressing the redox-inactive BCAT1 mutant enzyme inhibits osteoclast maturation

**Fig. 5:**
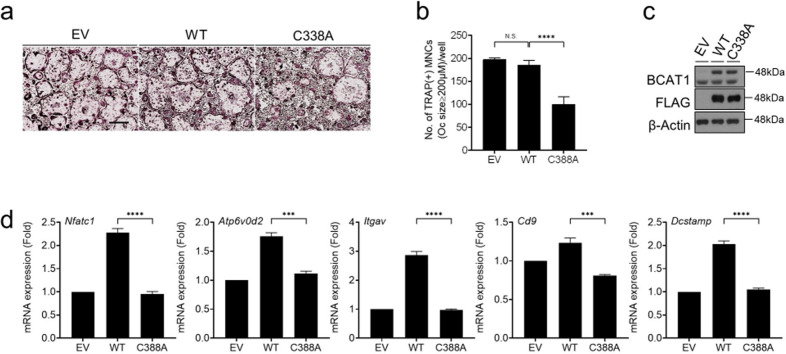
Expression of the inactive mutant of BCAT1 inhibits osteoclast maturation. BMMs were transduced with the empty pMX-puro retrovirus (EV), the retrovirus expressing 3xFlag-BCAT1 (WT) or the retrovirus expressing the inactive 3xFlag-BCAT1 mutant (C388A). The transduced BMMs were then cultured with M-CSF (30 ng/ml) and RANKL (100 ng/ml) for 4 days (**a**, **b**). The cells were stained for TRAP (**a**) and the TRAP^+^ cells with a diameter of ≥200 μm were counted (**b**). Scale bar, 500 μm. Total lysates of the cells were subjected to (**c**) western blot analysis to confirm DNA transduction or (**d**) RT-qPCR analysis of the indicated molecules. ***p ≤ 0.001, ****p ≤ 0.0001 (*n* = 4).

The mammalian BCAT enzymes both contain redox-active dithiol/disulfide Cys-Xaa-Xaa-Cys (CXXC) motif that allows them to be regulated by the redox state^24,25^. To confirm that BCAT1 can regulate osteoclast differentiation, we used site-directed mutagenesis to substitute cysteine 338 in the CXXC motif of BCAT1 with alanine (C338A): this mutation has been shown to block redox-mediated activation of the BCAT enzyme. When BMMs were transfected with a retrovirus that expressed either WT or C338A mutant BCAT1 and then subjected to the osteoclast differentiation, the mutant was found to significantly inhibit osteoclast differentiation (Fig. 5a–c). However, the mutant did not affect bone resorption activity (Supplementary Fig. 3d, e). The presence of the redox-inactive BCAT1 enzyme also associated with lower mRNA levels of the osteoclast maturation markers Atp6v0d2, Itgav, Cd9, and Dcstamp (Fig. 5d). These findings support the notion that BCAT1 regulates osteoclast maturation.

### Gabapentin inhibits LPS-induced bone destruction in mice

**Fig. 6:**
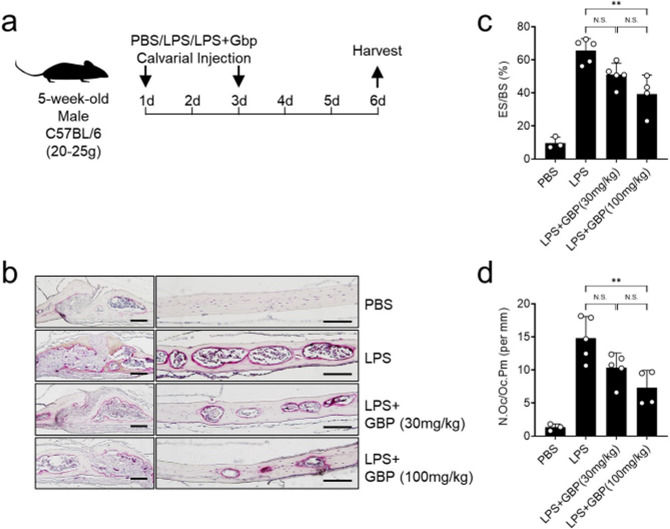
Gabapentin inhibits LPS-induced bone resorption in vivo. As depicted schematically (**a**), the calvaria of mice were injected twice with PBS, LPS (12.5 mg/kg of body weight) alone, or LPS with 30 or 100 mg/kg body-weight gabapentin. Three days after the last injection, the mice were sacrificed and their calvaria was sectioned and stained for TRAP (**b**). The amount of bone that was eroded was determined by histology and OsteoMeasucreXP analysis and was expressed as % eroded surface (ES)/bone surface (BS) (**c**). The number of osteoclasts per bone perimeter (N.Oc/B.Pm, per mm^2^) was also determined (**d**). *n* = 3, 5, 5 and 4 in the PBS, LPS alone, LPS + 30 mg/kg gabapentin, and LPS + 100 mg/kg gabapentin groups, respectively. **p ≤ 0.01. Scale bar, 50 μm.

To determine whether gabapentin treatment can reduce osteoporosis-like disease, we used the murine LPS-induced inflammatory bone destruction model. Thus, the calvaria of 5-week-old male mice was injected twice with either PBS, LPS alone, or LPS with 30 or 100 mg/kg body weight gabapentin (Fig. 6a). Gabapentin treatment reduced the LPS-induced bone surface erosions in a dose-dependent manner (Fig. 6b–c). It also reduced the number of osteoclasts (Fig. 6d). Thus, BCAT1 inhibition by gabapentin inhibited LPS-induced bone loss in mice. Notably, the gabapentin dose of 30 or 100 mg/kg body-weight that we used is in line with the concentrations in pre-clinical animal models for neuropathic pain (30–150 mg/kg) and seizures (≥300 mg/kg)^26–28^. Thus, gabapentin effectively inhibits bone destruction at acceptable concentrations.

## Discussion

High protein intake plays a significant role in the maintenance of bone health is not only in the growth phase but also in older people with osteoporosis^29,30^. Indeed, several studies suggest that high protein intake can help increase bone mineral density (BMD) and decrease bone loss and risk of hip fracture^31,32^. These observations together with the fact that excessive osteoclast activity can lead to skeletal loss and that osteoclasts have greater energy needs during differentiation led us to ask whether certain amino acids are involved in osteoclast formation or function. Indeed, we observed that the intracellular BCAA levels increase as RANKL-induced osteoclast differentiation progresses. Moreover, withdrawal of BCAAs at certain times during RANKL-induced osteoclast differentiation showed that these amino acids were essential for osteoclast maturation but not earlier differentiation stages. In addition, while all BCAAs were needed for osteoclast maturation, valine was the most important. Given that valine uptake was also the lowest of all three BCAAs, our results suggest that differentiating osteoclasts are particularly sensitive to changes in valine concentrations. Finally, when the BCAAs were replaced with their BCKA metabolites, osteoclast differentiation occurred normally.

These observations suggested that the BCATs that catalyze the transamination of BCAAs into BCKAs may participate in osteoclast differentiation and could be targeted therapeutically to ameliorate or prevent osteoclast-driven bone diseases. Indeed, we observed that while silencing BCAT2 did not affect osteoclast differentiation or resorption activity, inhibiting the cytosolic BCAT1 enzyme with gabapentin impaired both processes. Moreover, we also showed that BCAT1 enzymatic activity is needed for osteoclast differentiation since expressing an enzymatically inactive form of BCAT1 suppressed osteoclast formation. Importantly, we then found that LPS-induced bone erosion in mice was significantly decreased by concomitant treatment with gabapentin.

The latter findings are consistent with a recent study that indicated that BCAT1 regulates osteoclast maturation in vivo^33^. This study first showed that *Bcat1* is the hub gene of a *trans*-regulated gene network that regulates osteoclast differentiation and bone mass. The importance of *Bcat1* was then confirmed by knocking out *Bcat1* in mice: micro-CT analysis of the long bones revealed they had a greater bone mass and strength than the long bones of wild-type mice. Moreover, when *Bcat1* in human osteoclasts was knocked down by siRNA in vitro, they displayed reduced multinucleation^33^.

We observed that while a 400 μM solution of BCAAs efficiently promoted osteoclast differentiation, a higher concentration (800 μM) solution tended to inhibit it, suggesting that BCAAs have a negative feedback mechanism. This is supported by several studies. First, studies on cancer cells show that (i) BCAAs serve as strong nutrient signals and (ii) mainly act by activating mTORC1, which is a nutritional sensor that plays a key role in regulating cell growth, proliferation, and migration^34–36^. Second, Huynh et al. recently reported that mTORC1 hampers osteoclast differentiation via calcineurin and NFATc1. They also showed that while mTORC1 activity is high in osteoclast precursors, it is downregulated by RANKL stimulation. These observations together with our own suggest that BCAAs must be present at optimal levels or at an optimal BCAA ratio before osteoclasts can differentiate normally. This notion is supported by Duan et al., who reported recently that certain BCAA ratios enhance the proliferation and differentiation of myocytes and may activate the mTOTC1 nutrient sensor pathway in myocytes^37^. This in turn suggests that adequate BCAA levels and/or a balanced BCAA ratio in food may promote human and animal health.

BCAA levels are regulated by not only BCATs but also their principal transporter LAT1. Ozaki et al. showed recently that LAT1 regulates osteoclast differentiation^38^. In particular, they showed that when the gene that encodes LAT1 (*Slc7a5*) was deleted in only osteoclasts, the mice exhibited osteoclast activation and bone loss. However, since LAT1 also transports other neutral amino acids, it remains unclear whether the non-BCAA amino acids also shape bone homeostasis. Moreover, it is also possible that other transporters that regulate BCAA levels (e.g., LAT2, LAT4, and SNAT2) could influence osteoclasts and bone homeostasis in other ways. The roles of these transporters in osteoclast formation and bone metabolism remain to be determined.

The BCAAs are essential amino acids and account for up to 40% of the preformed amino acids that are required by mammals^39^. Essential amino acids in general have been shown to increase the BMD of older people^40^, and several cohort studies have shown that BCAA intake correlates positively with BMD^32,41^. Interestingly, one of these studies, which was on older patients (average age was 72 years), showed that high valine intake significantly inhibited BMD decline^32^. However, at present, we have little understanding of the molecular and cellular mechanisms that underpin these clinical observations.

Our study is the first to show that BCAA levels and the enzymatic activity of BCAT1 can regulate osteoclast formation and bone homeostasis. We therefore propose that BCAT1 may be a potential therapeutic target for osteoclast-associated bone diseases. However, further studies that elucidate the molecular mechanisms by which BCAT1 and BCAAs shape osteoclast maturation and bone metabolism are needed.

## Supplementary information

Supplementary Information
