# Supplementary material for: BCAT1 promotes osteoclast maturation by regulating branched-chain amino acid metabolism
Source: Exp Mol Med. 2022 Jun 27;54(6):825–33. doi: 10.1038/s12276-022-00775-3 (PMC9256685; doi:10.1038/s12276-022-00775-3)
Supplement: Supplementary file 1 — Supplementary Information [file 12276_2022_775_MOESM1_ESM.pdf]

Supplementary Information

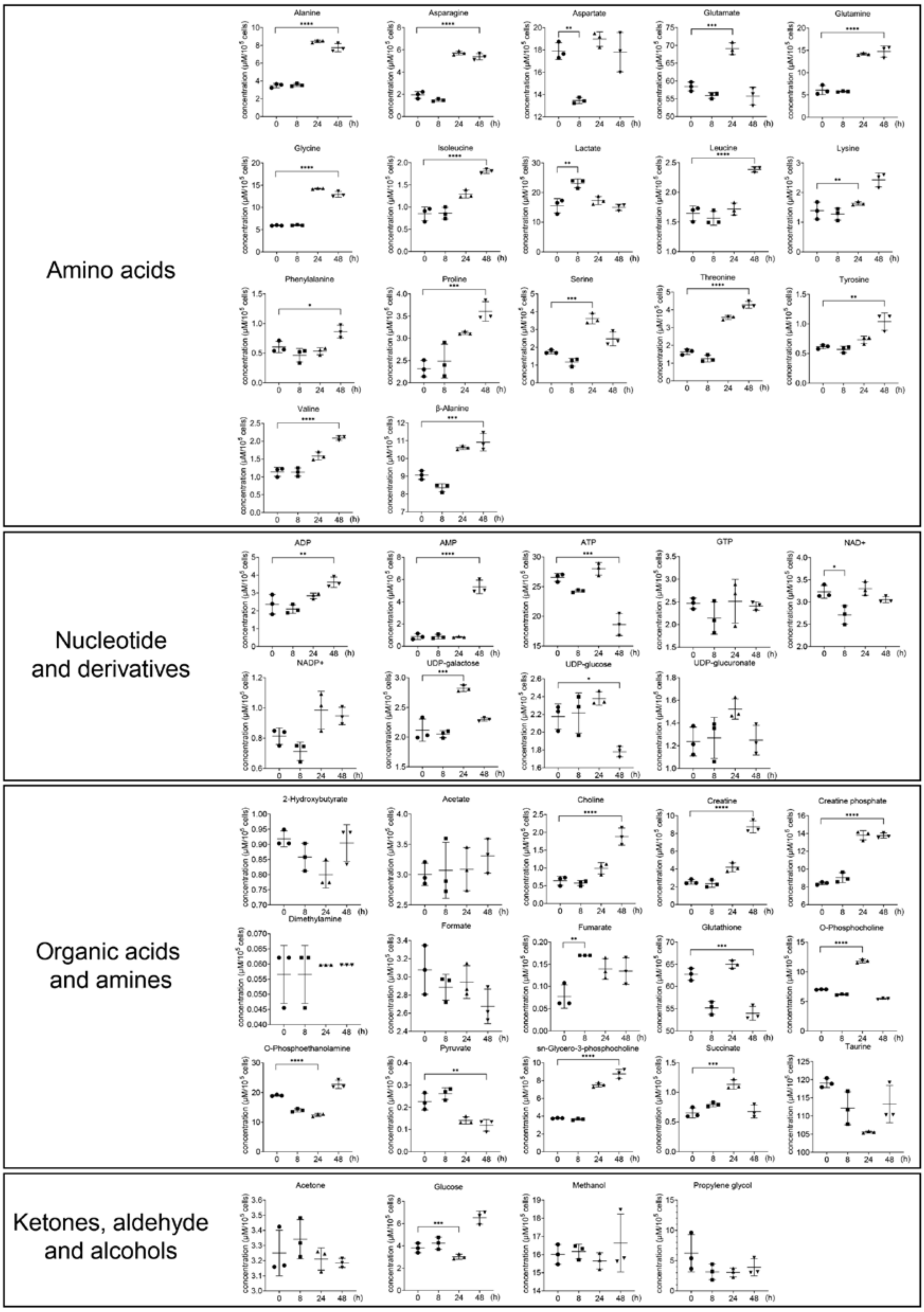

Supplementary Fig. 1. Change in metabolite levels during RANKL-induced osteoclast differentiation

BMMs were stimulated with RANKL (100 ng/ml) in alpha-MEM for the indicated times. The cells were then subjected to NMR spectroscopic analysis of the indicated metabolites (n=3). $^{*}p \leq 0.05$, $^{**}p \leq 0.01$, $^{***}p \leq 0.001$, $^{****}p \leq 0.0001$.

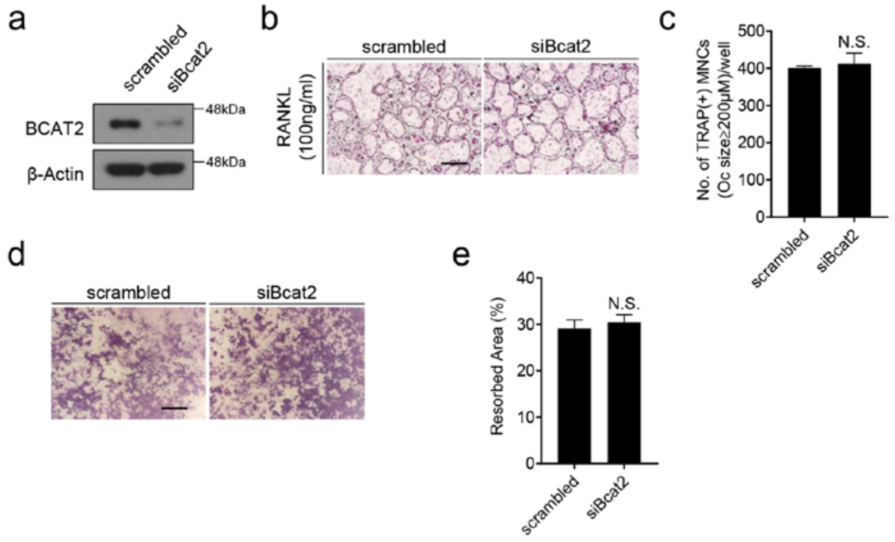

Supplementary Fig. 2. BCAT2 knockdown does not affect osteoclast differentiation. **(a-c)** BMMs were transfected with control siRNA (scrambled) or BCAT2 siRNA for 5 hours and cultured for 24 hours, after which the cells were treated with M-CSF (30 ng/ml) and RANKL (100 ng/ml) for 3 days. **(a)** The efficiency of siRNA knockdown was confirmed by western blotting with anti-Bcat2 antibody. **(b-c)** The siRNA-transfected osteoclasts were stained for TRAP **(b)**, after which $TRAP^+$ cells with a diameter of ≥200 μm were counted (n=4) **(c)**. **(d-e)** Alternatively, pre-osteoclasts were generated with RANKL (100 ng/ml) and transfected with siRNA. Transfected cells were transferred to dentin slices and further cultured with M-CSF (30 ng/ml) and RANKL (100 ng/ml) for 3 days. The resorbed pit area was visualized by staining with hematoxylin **(d)** and then quantified (n=4) **(e)**. Scale bar, 500 μm. N.S., not significant as determined by Student's *t*-test.

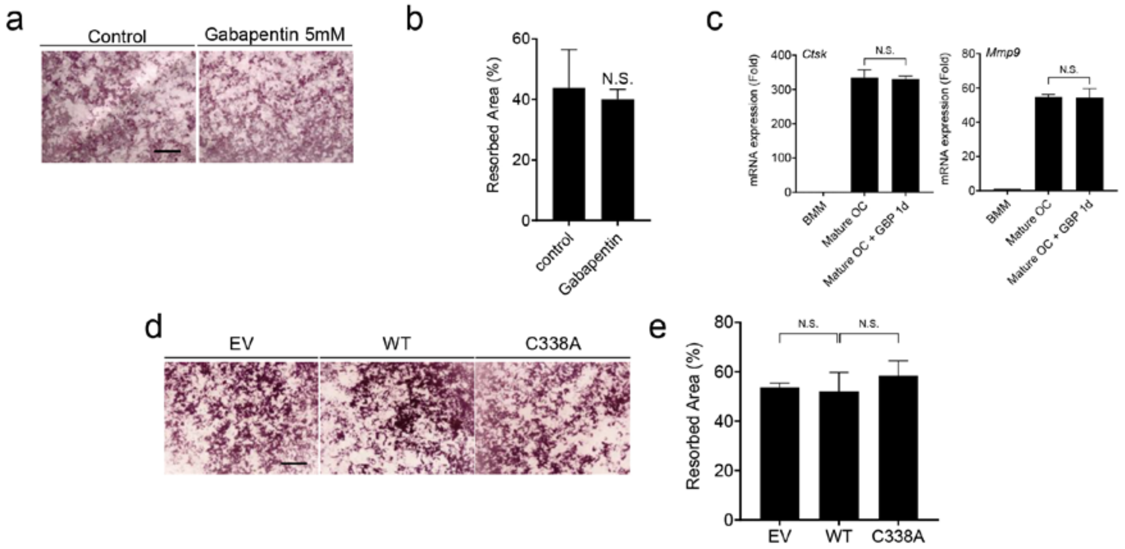

Supplementary Fig. 3. BCAT1 does not affect the bone-resorbing function of mature osteoclasts.

BMMs **(a-b)** or BMMs that were transfected with a retrovirus that expressed WT BCAT1 or the inactive C388A mutant of BCAT1 **(d-e)** were cultured with M-CSF (30 ng/ml) and RANKL (100ng/ml) for 2 days to generate pre-osteoclasts. The pre-osteoclasts were then transferred to dentin slices and further cultured with M-CSF and RANKL. **(a-b)** The transferred pre-osteoclasts were cultured with RANKL (100 ng/ml) alone or with gabapentin (5 mM) for 3 days **(a-b)**. The resorbed pit area was visualized by staining with hematoxylin **(a, c)**, and then quantified (n=4) **(b, d)**. **(c)** BMMs were cultured with M-CSF (30ng/ml) and RANKL (100ng/ml) for 4 days to generate mature multinucleated osteoclasts. The cells were then treated with gabapentin (5 mM) for 1 day. Scale bar, 500 μm. N.S., not significant as determined by Student's *t*-test.

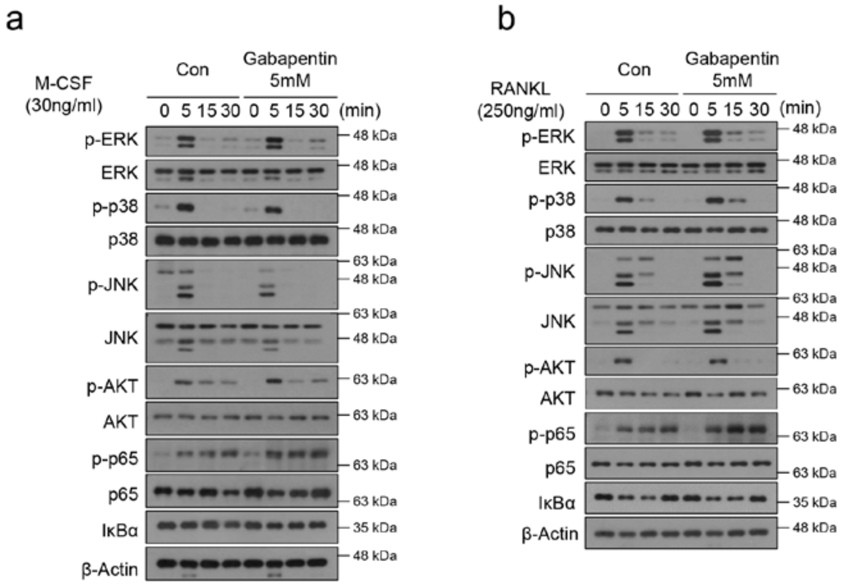

Supplementary Fig. 4. Gabapentin does not affect early M-CSF and RANKL signaling in osteoclast differentiation.

Serum-starved BMMs were preincubated with gabapentin (5 mM) for 2 hours and then stimulated with M-CSF (30 ng/ml) **(a)** or RANKL (250 ng/ml) **(b)** for the indicated times. The cells were subjected to western blot analysis with the indicated antibodies.
